# International Aid and Natural Disasters: A Pre- and Post-Earthquake Longitudinal Study of the Healthcare Infrastructure in Leogane, Haiti

**DOI:** 10.4269/ajtmh.14-0379

**Published:** 2015-02-04

**Authors:** Maxwell Kligerman, Michele Barry, David Walmer, Eran Bendavid

**Affiliations:** Stanford University School of Medicine, Stanford, California; Family Health Ministries, Durham, North Carolina; Center for Innovation and Global Health, Stanford School of Medicine, Stanford, California; Division of General Medical Disciplines, Stanford University, Stanford, California; Duke Global Health Institute, Durham, North Carolina; Center for Health Policy/Primary Care and Outcomes Research, Department of Medicine, Stanford University, Stanford, California

## Abstract

The reconstruction of healthcare systems in developing countries after natural disasters is poorly understood. Using data collected before and after the 2010 Haiti earthquake, we detail the response of aid agencies and their interaction with local healthcare providers in Leogane, the city closest to the epicenter. We find that the period after the earthquake was associated with an increase in the total number of healthcare facilities, inpatient beds, and surgical facilities and that international aid has been a driving force behind this recovery. Aid has funded 12 of 13 new healthcare facilities that have opened since the earthquake as well as the reconstruction of 7 of 8 healthcare facilities that have been rebuilt. Despite increases in free, aid-financed healthcare, private Haitian healthcare facilities have remained at a constant number. The planned phase-out of several aid-financed facilities, however, will leave Leogane with fewer inpatient beds and healthcare services compared with the pre-earthquake period.

## Background

As the amount of international aid spent annually on global health endeavors continues to increase, so too does controversy regarding aid effectiveness. Between 1990 and 2007, international aid spending for healthcare initiatives across the globe increased from $5.6 billion to $21.8 billion.^1^ Within recent years, the debate has intensified as to whether these initiatives are having their intended positive effect.^2^–^4^ Haiti, with its complicated history of aid funding and its strong non-governmental organization (NGO) presence, currently finds itself in the middle of this debate.^5^ In this study, we hope to shed light on the impact of international aid donors on healthcare provision through an analysis of the ongoing earthquake recovery effort in Haiti.

The January 12, 2010 Haiti earthquake killed 220,000 people, forced 1.5 million individuals into homelessness, and brought an entire country to its knees.^6^ The epicenter of the earthquake was less than 5 miles from Leogane, a coastal city 16 miles west of Port-au-Prince with an urban population of 50,000.^7^ Approximately 90% of the buildings in Leogane were either destroyed or severely damaged during the earthquake.^8^

After the earthquake, the international community launched one of the largest disaster relief efforts in modern history. Within weeks, over 17 field hospitals were established in Haiti, and the World Health Organization found itself coordinating over 400 healthcare providers in Haiti overall.^9^ During this period, the international community pledged billions of dollars in earthquake relief, and world leaders spoke optimistically about an opportunity for aid to help Haiti “build back better.”^10^

Empiric studies of the earthquake recovery process thus far have been sparse. Public records of healthcare facilities before the earthquake were largely incomplete, making a clear understanding of the impact of the earthquake and the ensuing recovery effort challenging.^11^ Some studies have focused on crude characterization of earthquake damage using either satellite imagery or data on the few registered public hospitals in the country.^12^ However, Haiti's healthcare system is a complex web of public, private-for-profit, and aid-financed facilities ranging in size from large hospitals to small community clinics, many of which were never registered with the government.^13^ Demographic and Health Surveys data provide an overview of health trends in Haiti; however, existing studies have failed to capture the detail necessary to show both the earthquake's impact on healthcare provision and the status of the recovery effort.^14^

The literature describing the impact of aid on local healthcare capacity in Haiti is also sparse. Some studies have described the flow of money, resources, and organizations into Haiti after the earthquake, but there has been little focus on the translation of these resource flows into healthcare provision.^15^,^16^ Speculative analysis suggested that the influx of medical aid may have unintended negative consequences on the community by competing or crowding out local healthcare providers.^17^–^19^ In some regions in Haiti, the provision of free healthcare has reduced patient flow to private healthcare facilities.^20^ However, the empiric data on this topic have also been limited.

Here, we study changes to the healthcare system in Leogane, Haiti and analyze the interaction between aid-financed organizations and the local healthcare system by using unprecedented pre- and post-earthquake data around a defined geographic area near the epicenter. Thus, for the first time, a longitudinal analysis is available to characterize the destruction from the earthquake and the cooperation that followed. Our findings provide new insights into the impact of international aid on the Haitian healthcare system and the coordination of local and aid-financed medical responses after a natural disaster.

## Methods

This study was coordinated by Family Health Ministries, a healthcare-focused non-profit organization that has been working in Haiti since 1993, and it was conducted in collaboration with the Stanford University School of Medicine and the Duke University Global Health Institute. Data collection was conducted by one of the authors (M.K.) and a community member from Leogane who helped to translate and guide.

We collected geocoded data of healthcare facilities and services in Leogane, Haiti at three time points: one time before the earthquake (June 1 to August 1, 2009) and two times after the earthquake (June 1, 2010 to January 10, 2011 and June 24 to August 1, 2013). We collected the initial data as part of a community needs assessment for Family Health Ministries, which at the time, was planning the construction of a new healthcare facility in Leogane. We conducted the two subsequent data collection waves to compare the results with our pre-earthquake data, recognizing the importance of characterizing the healthcare system's reconstruction.

During each wave of data collection, we logged the geographic coordinates of all healthcare facilities within a 25-mile^2^ area surrounding downtown Leogane by walking every road within this area (except for small footpaths without visible healthcare facilities). We considered a healthcare facility as any non-residential facility open to the public that has at least one registered physician, nurse, or optometrist. We used a list of all healthcare facilities registered with the Ministry of Health to verify our census.

We interviewed the onsite director of each healthcare facility using a standardized, semistructured questionnaire that included questions on the available services at the facility, the funding and income sources, and the healthcare personnel used in the facility. The survey instrument was developed by a panel of experts, including staff from Family Health Ministries and Duke University, and piloted before implementation. The complete survey is included in Supplemental Appendix.

We used the World Health Organization definition to classify hospitals as those healthcare facilities with inpatient capacity that are open 24 hours a day,^21^ and we classify outpatient clinics as those facilities with outpatient-only services. Because of a lack of standardization in describing funding patterns of healthcare facilities in Haiti, we created a classification system, whereby the degree of reported international aid was used to define facilities as local if they received no aid funding, collaborative if some but not all funding was provided by aid, and aid-financed if all of the funding was provided by international aid.

We used the same data collection methodology, survey instrument, community guide, and field researcher for all three waves of data collection.

## Findings

### Earthquake damage and healthcare facility closures.

**Figure 1.**
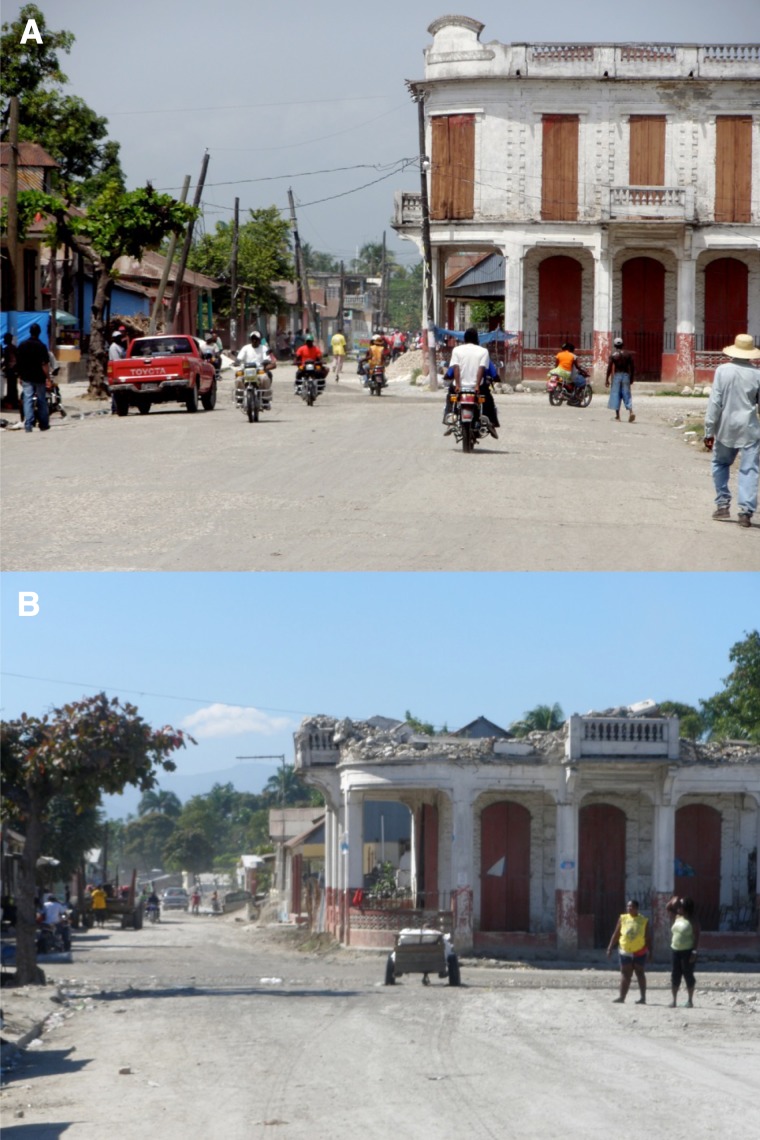
**A** and **B** demonstrate typical damage found throughout Leogane. (**A**) depicts one of the main thoroughfares of Leogane pre-earthquake. (**B**) depicts this same thoroughfare 1 year post-earthquake. Note the infrastructural damage, loss of electric poles, and general decrease in street activity.

Before the earthquake, in 2009, there were 25 healthcare facilities serving Leogane (21 outpatient clinics and 4 hospitals). During the January of 2010 earthquake, 11 of 25 healthcare facilities collapsed, including 8 outpatient clinics and 3 hospitals. Three facilities that collapsed (two hospitals and an outpatient clinic) functioned continuously after the earthquake but had reduced capacity. Six facilities that collapsed closed temporarily after the earthquake but reopened within 1 year. Two healthcare facilities that collapsed closed permanently, and one healthcare facility that did not sustain damage closed for unknown reasons. By 2011, 22 of 25 healthcare facilities functioning before the earthquake were again functioning; however, total inpatient beds among these facilities had declined from 232 to 124. Figure 1 presents pre- and post-earthquake scenes of Leogane, which are representative of the kind of damage found throughout the city.

### Post-earthquake growth of the healthcare system.

The period after the earthquake was associated with an increase in the number of both outpatient clinics and hospitals (Table 1). Between 2009 and 2011, the number of healthcare facilities increased from 25 to 28 (22 outpatient clinics and 6 hospitals). By 2013, the number of healthcare facilities serving Leogane increased to 34 (27 outpatient clinics and 7 hospitals).

The growth in the number of healthcare facilities was also associated with an increase in the number of operating rooms, the number of hospitals, the total number of inpatient beds, the number of facilities offering 24-hour emergency care, and the number of facilities offering primary healthcare services (Table 2). In 2009, there were 232 inpatient beds in 4 different hospitals, 1 facility had an operating room, there were no facilities offering 24-hour emergency medical services, 10 facilities provided infant immunizations, and 11 facilities provided pre-natal care. By 2011, there were 284 inpatient beds in 6 different hospitals, 4 facilities had operating rooms, 1 facility offered 24-hour emergency medical services, 12 facilities provided infant immunizations, and 14 facilities provided pre-natal care. By 2013, there were 342 inpatient beds in 7 different hospitals, 5 facilities had operating rooms, 1 facility offered 24-hour emergency medical services, 16 facilities offered infant immunizations, and 19 facilities offered pre-natal care. The number of facilities offering labor and delivery services remained the same.

### Contribution of aid to the rebuilding of the healthcare system.

**Figure 2.**
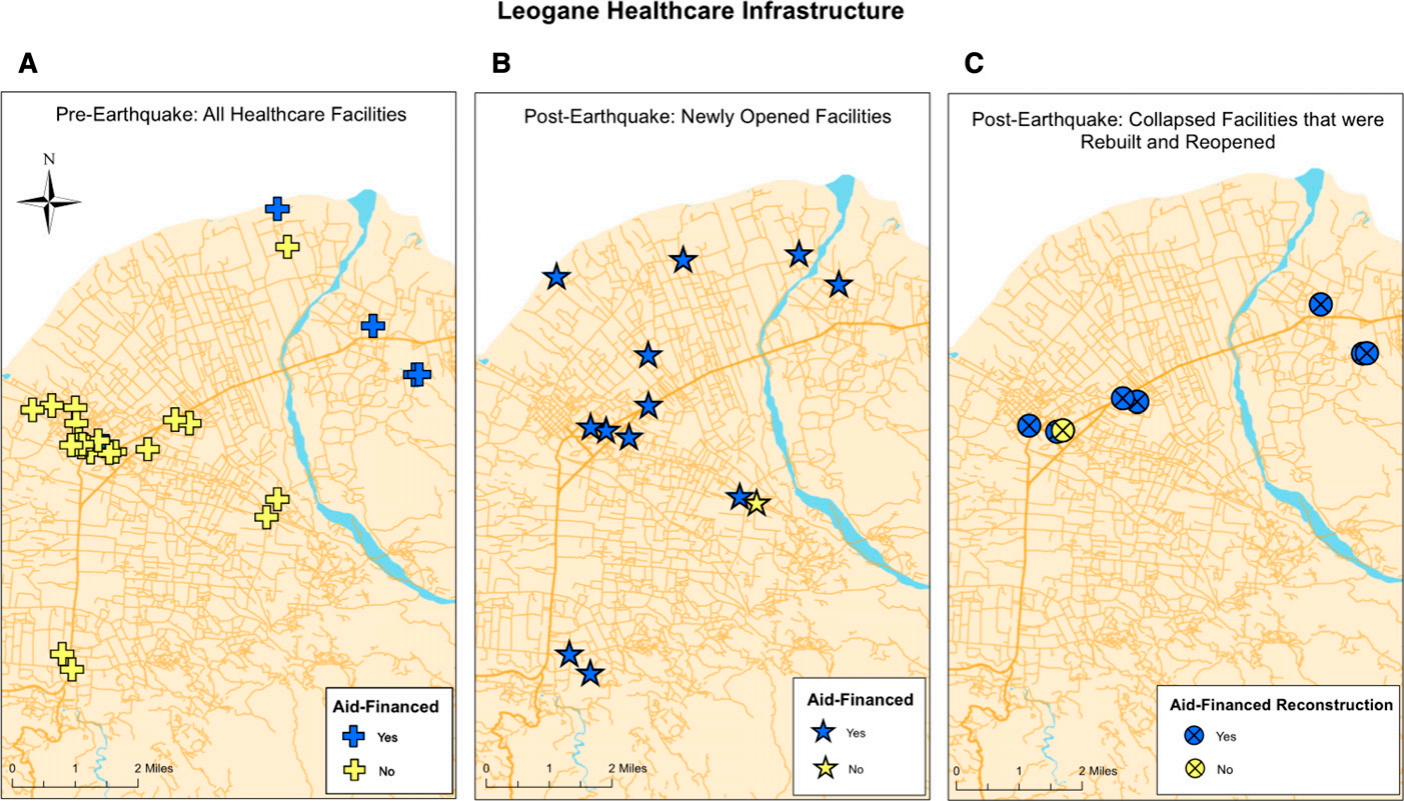
Leogane healthcare infrastructure. (**A–C**) Demonstrate the role of international aid on Leogane?s healthcare infrastructure both pre and post-earthquake. (**A**) Depicts the funding source of all healthcare facilities functioning in Leogane prior to the earthquake and demonstrates that aid-financed healthcare constituted a minority of facilities. (**B**) Depicts funding for healthcare facilities that opened post-earthquake and demonstrates that twelve of thirteen new healthcare facilities were aid-financed. (**C**) Depicts funding for the reconstruction of collapsed healthcare facilities and demonstrates that seven of eight healthcare facilities were able to reopen via aid-financed reconstruction.

The total number of aid-financed healthcare facilities increased from 5 in 2009 to 9 in 2011 to 13 in 2013 (Table 3). All aid-financed healthcare facilities were operated and funded by international NGOs. Before the earthquake, aid-financed healthcare facilities constituted a minority of all healthcare facilities in Leogane (Figure 2A), and there were no aid-financed healthcare facilities with inpatient capacity. However, 12 of 13 new healthcare facilities that opened after the earthquake were aid-financed healthcare facilities (Figure 2B). After the earthquake, Medicine Sans Frontiers (MSF) opened the largest inpatient facility in Leogane, which has 160 inpatient beds, two operating rooms, and 24-hour emergency medical services. The MSF facility has become the main referral hospital for all of Leogane and is largely responsible for the increase in inpatient capacity after the earthquake.

Aid also funded the reconstruction of healthcare facilities damaged in the earthquake. Seven of eight healthcare facilities that collapsed and reopened received aid to fund reconstruction (Figure 2C). Aid contributions also helped Hospital St. Croix, historically Leogane's largest hospital, to reopen as a collaborative healthcare facility after years of closure.^22^ Also during this time period, two international organizations collaborated with the Haitian Ministry of Health to open seven outpatient primary care clinics located in underserved areas of Leogane outside the city center.

### Hiring practices for aid-financed and collaborative healthcare facilities.

As of 2013, 8 of 13 aid-financed healthcare facilities had only Haitian healthcare providers, 4 facilities had a mixed staff of Haitian and international healthcare providers, and exact data on hiring practices for 1 facility (MSF) were unavailable. However, worldwide, 92% of MSF field staff are locally hired national employees.^23^ Seven of eight collaborative healthcare facilities use only Haitian healthcare providers, whereas one facility employs a mixed international and Haitian staff. No healthcare facility in Leogane used only international healthcare providers at the time of the 2013 data collection.

### Availability of free care and competition for private healthcare facilities.

The total number of healthcare facilities in Leogane offering free medical services (treatment and medication) has increased each year from zero in 2009 to four in 2011 to seven by 2013. All of the facilities that offer free medical services are aid-financed healthcare facilities. Despite this increase in free medical services, the total number of healthcare facilities that are privately owned has remained steady. Between 2009 and 2013, the number of private facilities has remained steady at 13, with 2 private facilities closing, 1 private facility becoming a collaborative facility, 2 facilities privatizing, and 1 new private facility opening. As of 2013, 2 of 13 private healthcare facilities were hospitals (one of which offered surgical services), and the remaining 11 facilities were outpatient clinics offering primary care-type services. The types of primary care services offered by private healthcare facilities have exhibited some fluctuation, but taken as a whole, the data show relative consistency. Since 2009, the number of private facilities offering labor and delivery services, for example, has remained steady at four; the number offering infant immunizations has decreased from seven to five, but the number offering pre-natal care has increased from eight to nine (Supplemental Tables 1–4). The price for a consultation at all private facilities in Leogane has also increased from an average of 115 Haitian Gourdes (approximately $2.50 US) in 2009 to 172 Gourdes (approximately $3.75 US) in 2013. Directly comparing just those private facilities that were functioning in both 2009 and 2013 shows a slightly greater price increase from 125 Haitian Gourdes (approximately $2.70 US) in 2009 to 183 Haitian Gourdes (approximately $4.00 US) in 2013 (Supplemental Tables 5 and 6).

Despite the consistent number of private facilities in Leogane, the overall number of local healthcare facilities has exhibited a slight decline from 14 in 2009 to 13 in 2013. The number of healthcare facilities operated by the Ministry of Health has remained steady at two throughout the duration of this study.

### The transition and closure of aid-financed facilities.

Between 2009 and 2013, five aid-financed outpatient healthcare facilities withdrew from Leogane, with two facilities transitioning into a collaborative partnership and three facilities closing. In December of 2015, MSF plans to close its Leogane facility. Without these 160 inpatient beds and in the absence of increased capacity from other sources, Leogane will have 22% fewer inpatient beds than were present before the earthquake. Additionally, with the departure of MSF, 24-hour emergency medical services will again be unavailable. Within the next several years, at least five additional aid-financed healthcare facilities are intended to transfer to the authority of the Ministry of Health.

## Discussion

Our findings show that the healthcare infrastructure of Leogane, the city closest to the epicenter of the 2010 Haiti earthquake, suffered extensive damage, because nearly one-half of all healthcare facilities collapsed. However, by 2013, healthcare services in Leogane were more abundant, affordable, and comprehensive than immediately before the earthquake. The numbers of outpatient clinics, hospitals, inpatient beds, free care facilities, 24-hour emergency facilities, and surgical centers have all increased from pre-earthquake levels. The addition of seven primary care clinics in previously underserved areas has increased access to preventive care, and the fivefold increase in surgical facilities has likely increased access to specialty services.

Our findings show the critical role played by aid in the rebuilding process. Although some sources argue that international aid agencies have made disappointingly little progress in the recovery effort, we find that aid agencies have substantially expanded Leogane's healthcare system and financially supported nearly every aspect of the recovery process.^24^–^26^ Nearly all collapsed healthcare facilities were rebuilt within 1 year with aid-financed support. In addition, 12 of 13 new healthcare facilities that have opened since the earthquake have been aid-financed. We attribute aid's successful impact in Leogane to several factors. First, aid organizations focused efforts on the construction of multiple small inexpensive healthcare facilities as opposed to a small number of more intricate and expensive facilities. This approach has helped make the transition of care to local capacity much easier and allowed aid to couple short-term earthquake relief with long-term increases in healthcare capacity. Second, at least 11 different aid organizations were already working in Leogane at the time of earthquake. These pre-existing institutions galvanized extra support for the relief effort and provided a pre-established infrastructure to help facilitate aid delivery. Third, the small size of Leogane and its healthcare system enabled one large facility, like MSF, to completely transform the landscape and improve every aspect of healthcare delivery in the community.

In other contexts, the influx of aid-financed goods and services has undermined local capacity and profits.^27^,^28^ This was a concern in Leogane, because before the earthquake, 50% of healthcare facilities were private practices. However, our data indicate that the number of private healthcare facilities has remained stable during the accompanying increase in free facilities. Although offering free medical services for extended periods may undercut profits for local private facilities,^29^ we find little evidence for closure of private clinics in response to the growth in aid-financed health services. Local private capacity has remained stable in large part because aid facilities have offered services that were previously unavailable and do not overlap with local capacity, such as specialized tertiary care, surgical services, and emergency services. Although some aid organizations do also offer primary healthcare services, data on the availability of these services corroborate that labor and delivery, infant immunizations, and pre-natal care services have not shifted away from private facilities. This is likely because newly opened aid-financed primary care facilities have been built far away from any pre-existing private facilities offering similar services. Also, many patients seem to have retained a preference for the shorter waits and more focused care available at private facilities. The price for patient consultations at private healthcare facilities has also increased since the earthquake, enabling facilities to continue to function despite fewer patient visits.

The rebuilding process in Leogane is at a crossroads. With the pending departure of aid agencies, Leogane's healthcare infrastructure is at risk of regressing. Without transitioning of capacity, Leogane will have fewer inpatient beds than before the earthquake and no 24-hour emergency medical services. The growing dependence on aid is suggested by the decrease in the number of non-aid–financed inpatient beds and the slight decrease in the number of local healthcare facilities. Although the growing interconnectedness between international and local healthcare facilities does have many positive aspects, it potentially leaves Leogane's healthcare system vulnerable to any sudden reductions in aid. Using Leogane to generalize on the balance of healthcare across Haiti underscores the need for aid agencies to more deliberately plan transitions and build local capacity.

The pending departure of MSF, in particular, will profoundly change the healthcare system in Leogane. Although MSF has played a crucial role in Leogane's recovery, we suspect that it has likely stalled the development of new local and collaborative healthcare facilities that have been unable to compete for patients. Therefore, we theorize that the departure of MSF will result in a transient decrease in healthcare services followed quickly by an increase in local and collaborative capacity. Specific specialty services, however, will likely remain unavailable for a longer period. It will be important to study how the withdrawal of MSF will change healthcare delivery in Leogane and assess any associations between the withdrawal and an increase in the rate of growth of permanent healthcare capacity. It will also be critical to continue to monitor healthcare needs in the rural towns surrounding Leogane, because MSF was the safety net hospital for much of this region.

This study is limited in its generalizability to other natural disaster situations, although the themes of aid effectiveness and dependency are familiar from other contexts. This study is also limited in its ability to distinguish between specific budgets or funding routes of aid providers. It is also important to note that this study focuses on tracking healthcare facilities rather than individual healthcare providers. Therefore, although we have determined that facilities have not been crowded out and forced to close, we are unable to determine if the overall number of physicians serving Leogane has increased, decreased, or remained the same. Similarly, although we have determined the number of healthcare facilities offering specific primary healthcare services, we are unable to determine if the frequency with which each facility performed these services has changed. Lastly, although the researchers recognize the unique role played by faith-based organizations in Haiti's healthcare system, an analysis of this sector falls outside the scope of this paper.

In light of the ongoing debate regarding the positive and negative aspects of international aid in Haiti, we hope our findings show that aid has been a crucial driving force behind Leogane's healthcare system recovery. Looking forward, we intend to qualitatively study how healthcare providers in Leogane view the impact of aid. Thus far, we have found that healthcare providers in Leogane had positive perceptions of international aid, including acute emergency relief and long-term improved healthcare access; however, they also identified several negative impacts of aid, like episodes of poor quality care, competition across facilities, and a decrease in patient flow to local facilities. These themes need to be further explored, and it will also be essential to study patients' perceptions of aid to better address specific healthcare concerns. Overall, it will be important to continue to monitor Leogane in the years to come to understand long-term impacts of aid, assess the impact of aid withdrawal, and continue to learn best practices for healthcare system recovery.

## Supplementary Material

Supplemental Datas.

## Figures and Tables

**Table 1:** Number of healthcare facilities by year

	2009	2011	2013
Outpatient clinics	21	22	27
Hospitals	4	6	7
Total	25	28	34

**Table 2:** Types of medical services available by year

	2009	2011	2013
Inpatient beds	232	284	342
Facilities with an operating room	1	4	5
Facilities with free medical services	0	4	7
Facilities providing infant immunizations	10	12	16
Facilities providing pre-natal care	11	14	19
Facilities providing labor and delivery services	7	8	7

**Table 3:** Healthcare facility funding by year

	2009	2011	2013
Aid-financed facilities	5	9	13
Collaborative facilities	6	5	8
Local facilities	14	14	13

## References

[1] Ravishankar N, Gubbins P, Cooley RJ, Leach-Kemon K, Michaud CM, Jamison DT, Murray CJ (2009). Financing of global health: tracking development assistance for health from 1990 to 2007. Lancet.

[2] Ranis G (2011). Giving up on foreign aid?. Cato J.

[3] David Skarbek PL (2009). What can aid do?. Cato J.

[4] Lu C, Schneider MT, Gubbins P, Leach-Kemon K, Jamison D, Murray CJ (2010). Public financing of health in developing countries: a cross-national systematic analysis. Lancet.

[5] Zanotti L (2010). Cacophonies of aid, failed states building and NGOs in Haiti: setting the state for disaster, envisioning the future. Third World Q.

[6] Office for the Coordination of Humanitarian Affairs (2011). Haiti Earthquake Reponse.

[7] Lammie PJ, Walmer D (2014).

[8] Eberhard MO, Baldridge S, Marshall J, Mooney W, Rix GJ (2010). The Mw 7.0 Haiti Earthquake of January 12, 2010: USGS/EERI Advance Reconnaissance Team Report.

[9] PAHO/WHO (2011). Earthquake in Haiti–One Year Later, PAHO/WHO Report on the Health Situation.

[10] United Nations Office of the Special Envoy for Haiti (2010). Summary Report on Activities, June 2009–December 2010.

[11] Barnes-Josiah D, Myntti C, Augustin A (1998). The “three delays” as a framework for examining maternal mortality in Haiti. Soc Sci Med.

[12] Eguchi RT, Shubharoop Ghost SPG, Svekla W, Adams BJ, Evans G, Toro J, Saito K, Spence R (2010). The January 12, 2010 Haiti earthquake: a comprehensive damage assessment using very high resolution areal imagery.

[13] Kelly A, Roberts J (2011). Is Haiti's Health System Any Better? A Report Calling for a More Coordinated, Collaborative Approach to Disaster Response.

[14] Ministry of Public Health and Population; Haitian Childhood Institute; ICF International (2012). Haiti 2012 Mortality, Morbidity, and Service Utilization Survey Key Findings.

[15] United Nations Office of the Special Envoy for Haiti (2011). Has Aid Changed? Channeling Assistance to Haiti Before and After the Earthquake.

[16] United Nations Office of the Special Envoy for Haiti (2012). Can More Aid Stay in Haiti and Other Fragile Settings? How Local Investment Can Strengthen Governments and Economies.

[17] Biquet J-M, Policy ID (2013). Haiti: Between Emergency and Reconstruction.

[18] Adams P (2010). Health-care dynamics in Haiti. Lancet.

[19] Jobe K (2011). Disaster relief in post-earthquake Haiti: unintended consequences of humanitarian volunteerism. Travel Med Infect Dis.

[20] Haar RJ, Naderi S, Acerra JR, Mathias M, Alagappan K (2012). The livelihoods of Haitian health-care providers after the January 2010 earthquake: a pilot study of the economic and quality-of-life impact of emergency relief. Int J Emerg Med.

[21] World Health Organization (2014). Hospitals.

[22] Bessinger CD, McNeeley DF (1984). A cooperative model for provision of regional health services in a developing nation. JAMA.

[23] Hudood B (2013). Without Borders.

[24] Arie S (2012). Work of 125 aid agencies failed to create lasting rehabilitation services in Haiti, study shows. BMJ.

[25] Sontag D (2012). Rebuilding in Haiti lags after billions in post-quake aid. New York Times.

[26] Waltz VRAJ (2012). Haiti: Where Has All the Money Gone?.

[27] Deaton A (2013). The Great Escape: Health, Wealth, and the Origins of Inequality.

[28] Benjamin E, Bassily-Marcus AM, Babu E, Silver L, Martin ML (2011). Principles and practice of disaster relief: lessons from Haiti. Mt Sinai J Med.

[29] Green A (1987). The role of non-governmental organizations and the private sector in the provision of health care in developing countries. Int J Health Plann Manage.

