# Supplementary material for: International Aid and Natural Disasters: A Pre- and Post-Earthquake Longitudinal Study of the Healthcare Infrastructure in Leogane, Haiti
Source: Am J Trop Med Hyg. 2015 Feb 4;92(2):448–53. doi: 10.4269/ajtmh.14-0379 (PMC4347354; doi:10.4269/ajtmh.14-0379)
Supplement: Supplementary file 1 [file SD11.pdf]

SUPPLEMENTAL APPENDIX

Interview guide in 2009.

(1) What is the healthcare facility's name?

(2) What is the primary patient population (women, children, family, subspecialty, etc.)?

(3) Inpatient, outpatient, both? If inpatient, how many beds are present?

(4) Operating room present?

(5) Hours of operation?

(6) Price for a consultation?

(7) Number of doctors? Specialties?

(8) Number of nurses?

(9) Pharmacy present?

(10) How is the facility funded?

(11) Is there external aid? If so, from which organization?

(12) Approximately how many patients per month does the facility serve?

(13) Are any of the following women's health services available: labor/delivery, pre-natal care, infant immunizations, birth control?

(14) Are any of the following women's surgical services available: C-section, hysterectomy, ovarian cyst removal, tubal ligation?

(15) What other surgical services are available?

(16) What are the biggest operational challenges for the clinic?

(17) What is the largest health issue in the community?

Additional questions in 2010 and 2011.

(18) Did the healthcare facility suffer any damage in the earthquake?

(19) How have healthcare services changed since the earthquake?

(20) How has funding and international aid changed since the earthquake?

Additional questions in 2013.

(18) When was the healthcare facility established?

(19) Would you identify this clinic as a local initiative, an international initiative, or a collaboration?

(20) What proportion of physicians are non-Haitian versus Haitian?

(21) How has aid changed since the earthquake?

(22) Were there any kinds of aid received that were particularly helpful or unhelpful?

(23) From your perspective, what are some of the positive and negative aspects of the influx in international healthcare providers to Leogane?

(24) How has your practice been impacted by the influx of international aid and international organizations?

(25) How is your relationship with non-Haitian/international healthcare facilities?

(26) How is your relationship with local Haitian/local healthcare facilities?

(27) What is the long-term plan for the facility (i.e. close, move to new location, expand, maintain status quo, etc.)?

Supplemental Table 1

All healthcare facilities in Leogane

Facility name	Facility type	Operator	Funding	Status 2009	Status 2011	Status 2013	Earthquake impact
Fosref	Clinic	NGO	Aid-financed	Open	Closed	Closed	Collapse
New Missions Clinique	Clinic	NGO	Aid-financed	Open	Closed	Closed	Collapse
Haiti Health Ministries Clinic	Clinic	NGO	Aid-financed	Open	Open	Open	Collapse
Clinique de Dezman Fleury	Clinic	NGO	Aid-financed	Open	Open	Open	Collapse
Christianville Eye Clinic	Clinic	NGO	Aid-financed	Open	Open	Open	Collapse
Hermann Lunitierre	Clinic	Private	Local	Open	Open	Closed	Collapse
Sama Plus*	Clinic	Collaborative/private	Collaborative/local	Open	Open	Open	Collapse
Clinique Medicale	Clinic	Private	Local	Open	Closed	Closed	No damage
Laboratoire Medical Bon Berger	Clinic	Private	Local	Open	Open	Open	No damage
Center de Prosch	Clinic	Private	Local	Open	Open	Open	No damage
Center Materno Infantil	Clinic	Ministry of Health	Local	Open	Open	Open	No damage
Clinique des Enfants	Clinic	Private	Local	Open	Open	Open	No damage
Clinqiue Centro de Sante	Clinic	Private	Local	Open	Open	Open	No damage
Bethesda Health Center	Clinic	Private	Local	Open	Open	Open	No damage
Center de Sante Darbon	Clinic	Private	Local	Open	Open	Open	No damage
Macaya Lib	Clinic	Private	Local	Open	Open	Open	No damage
Clinique de Puple	Clinic	Private	Local	Open	Open	Open	No damage
Center de Sante Marie Jean†	Clinic	Private/collaborative	Local/collaborative	Open	Open	Open	No damage
Sant Materno Infantil de Timo*	Clinic	Collaborative/private	Collaborative/local	Open	Open	Open	No damage
Comejo‡	Clinic/hospital	Private	Local	Open	Open	Open	No damage
Hopital St. Croix§	Clinic/hospital	Collaborative	Collaborative	Open	Open	Open	Collapse
Help Hospital	Hospital	Collaborative	Collaborative	Open	Open	Open	Collapse

(continued)

Supplemental Table 1
Continued

Facility name	Facility type	Operator	Funding	Status 2009	Status 2011	Status 2013	Earthquake impact
Hopital Cardinal Leger	Hospital	Collaborative	Collaborative	Open	Open	Open	Collapse
Sanatorum	Hospital	Ministry of Health	Collaborative	Open	Open	Open	Collapse
Center Hospitalier Hosanna	Hospital	Private	Local	Open	Open	Open	No damage
Malteser International¶	Clinic	NGO/ collaborative	Aid-financed/ collaborative	Not yet open	Open	Open	N/A
Klinik Kominite Lasante¶	Clinic	NGO/ collaborative	Aid-financed/ collaborative	Not yet open	Open	Open	N/A
International Medical Corp Clinic	Clinic	NGO	Aid-financed	Not yet open	Open	Closed	N/A
Heart to Heart Headquarters Clinic	Clinic	NGO	Aid-financed	Not yet open	Open	Open	N/A
The Johannitor Headquarters Clinic	Clinic	NGO	Aid-financed	Not yet open	Open	Open	N/A
Doctors Without Borders	Hospital	NGO	Aid-financed	Not yet open	Open	Open	N/A
Heart to Heart Clinic 1	Clinic	NGO	Aid-financed	Not yet open	Not yet open	Open	N/A
Heart to Heart Clinic 2	Clinic	NGO	Aid-financed	Not yet open	Not yet open	Open	N/A
Johannitor Clinic 1	Clinic	NGO	Aid-financed	Not yet open	Not yet open	Open	N/A
Johannitor Clinic 2	Clinic	NGO	Aid-financed	Not yet open	Not yet open	Open	N/A
Johannitor Clinic 3	Clinic	NGO	Aid-financed	Not yet open	Not yet open	Open	N/A
Johannitor Clinic 4	Clinic	NGO	Aid-financed	Not yet open	Not yet open	Open	N/A
Johannitor Clinic 5	Clinic	NGO	Aid-financed	Not yet open	Not yet open	Open	N/A
St. John Babstiste Center de Sante Darbon	Clinic	Private	Local	Not yet open	Not yet open	Open	N/A

N/A = not applicable.
*Collaborative in 2009; local in 2011 and 2013.
†Local in 2009; collaborative in 2011 and 2013.
‡Clinic in 2009; hospital in 2011 and 2013.
§Clinic in 2009 and 2011; hospital in 2013.
¶Aid-financed in 2011; collaborative in 2013.

Supplemental Table 2
Number of facilities offering labor and delivery services

	2009	2011	2013
Aid-financed	1	3	1
Collaborative	2	1	2
Local*	4	4	4

* Local facilities offering labor and delivery services are all private facilities.

Supplemental Table 3
Number of facilities offering infant immunizations

	2009	2011	2013
Aid-financed	0	2	5
Collaborative	2	3	5
Local*	8	7	6

*Local facilities offering infant immunizations include one local public facility; all others are private.

Supplemental Table 4
Number of facilities offering pre-natal care

	2009	2011	2013
Aid-financed	2	4	6
Collaborative	1	3	3
Local*	8	7	10

*Local facilities offering pre-natal care in 2009 and 2011 are all private facilities. In 2013, local facilities include one local public facility.

Supplemental Table 5

Consultation price for all private facilities (in Haitian Gourdes)

Facility name	Facility type	Consultation price 2009	Consultation price 2013
Clinique Medicale	Private clinic	150	–
Laboratoire Medical Bon Berger	Private clinic	250	500
Center de Prosch	Private clinic	100	200
Clinique des Enfants	Private clinic	100	150
Clinqiue Centro de Sante	Private clinic	100	
Bethesda Health Center	Private clinic	100	50
Center de Sante Darbon	Private clinic	100	100
Clinique de Puple	Private clinic	100	100
Center de Sante Marie Jean	Private/collaborative clinic	50	–
Sant Materno Infantil de Timo	Collaborative/private clinic	–	375
Comejo Polyclinic	Private clinic/hospital	–	50
St. John Babstiste Center de Sante Darbon	Clinic	–	25

Supplemental Table 6

Consultation price for healthcare facilities functioning in both 2009 and 2011 (in Haitian Gourdes)

Facility name	Facility type	Consultation price 2009	Consultation price 2013
Laboratoire Medical Bon Berger	Private clinic	250	500
Center de Prosch	Private clinic	100	200
Clinique des Enfants	Private clinic	100	150
Bethesda Health Center	Private clinic	100	50
Center de Sante Darbon	Private clinic	100	100
Clinique de Puple	Private clinic	100	100
